# Neurocognitive and Neurological Effects of Coffee and Caffeine: A Narrative Review

**DOI:** 10.7759/cureus.94742

**Published:** 2025-10-16

**Authors:** Joseph V Pergolizzi, Julius T Tenenbaum, Claudio Pergolizzi, Jo Ann K LeQuang

**Affiliations:** 1 Executive Department, NEMA Research, Inc., Naples, USA; 2 Pain Management Department, NEMA Research, Inc., Naples, USA; 3 Scientific Operations Department, NEMA Research, Inc., Naples, USA; 4 Scientific Communications Department, NEMA Research, Inc., Naples, USA

**Keywords:** adenosine antagonism, alzheimer's disease, caffeine, coffee, cognitive improvement, neuroprotection, parkinson's disease, polyphenols

## Abstract

Coffee, a popular beverage, is surprisingly complex and increasingly studied for its potential health benefits. In particular, coffee contains caffeine, which may play a role in alertness, cognition, and memory. Coffee also contains an abundance of polyphenols and other compounds, which may confer specific health benefits. It is well known that coffee drinking can ward off drowsiness and fatigue, but the mechanisms behind this are not clearly understood. The caffeine in coffee can inhibit adenosine, but this mechanism is not well understood. Whether or not coffee has protective effects against neurodegenerative conditions is being actively studied. Coffee may confer significant neuroprotective, anti-inflammatory, and cognitive benefits, which are of particular interest in aging populations and warrant greater study. A challenge in studying coffee's neurocognitive aspects is that there are multiple types of coffee, and "dosing," or how much coffee is consumed over time, can vary widely among studies.

## Introduction and background

Regular coffee drinkers have lower rates of several neurodegenerative conditions, including Parkinson’s disease and Alzheimer’s disease [1,2], dementia [3], stroke [1], and multiple sclerosis [2,3]. However, the complexity of coffee, along with cultural consumption patterns and genetics, makes it difficult to clearly and unequivocally attribute these benefits to specific coffee compounds or a synergistic interplay of certain constituents.

While the main and best-known constituents of coffee are caffeine and other purine metabolites, such as theobromine, theophylline, and paraxanthine [4], the role of caffeine in neuronal plasticity and synaptic development is not well studied [5]. Neuroplasticity refers to the brain's ability to form new neural connections either by altering synaptic structure in synaptic plasticity or growing new neurons in neurogenesis. Caffeine can modulate receptors and affect channel activities in the brain, notably inhibiting phosphodiesterase, adenosine, and gamma-aminobutyric acid (GABA) cellular receptors [6]. However, other caffeinated beverages do not seem to have the same effects as coffee.

With an aging global population, the neuroprotective aspects of coffee against neurodegenerative conditions are of great interest. Animal studies show that coffee and/or caffeine confer neurologic benefits and may improve memory, attention, and cognition [7]. However, coffee, like other natural substances, can be challenging to study because there are many different varieties of beans, various processing and roasting techniques, numerous ways of serving the beverage, and a range of consumption patterns (“dose”), any of which may impact its effects on the brain. The purpose of this narrative review is to examine the main constituents of coffee, their potential roles in neuroprotection, neuroplasticity, cognition, and analgesia, as well as to review the relevant clinical studies conducted to date.

Methods

This was a narrative review, which means we had a research topic rather than a specific research question. This meant our literature search was broad but not particularly deep. Coffee has not been investigated systematically, and the studies we found were heterogeneous, preventing us from formulating a single research question to develop a systematic review. Nevertheless, the topic is of sufficient interest to provide a narrative review of what is currently known.

An initial search used the PubMed database to find randomized clinical trials and clinical trials (in humans only) published in the past 10 years, using the broad keyword "coffee + brain." This yielded 15 results. Since these results were from clinical trials, including randomized clinical trials, we considered them particularly important and summarized them in tabular format. However, these studies were heteorgenous; thus, they could not be systematically synthesized.

A broader search using PubMed was conducted for the keywords "coffee + neuroplasticity" (n=16), "neuroplastic effects + coffee" (n=13), and "caffeine neuroplasticity" (n=98). This produced 127 results, of which 15 were duplicates and 40 were excluded because they were not specifically relevant to coffee. This resulted in 72 papers of various types. This included articles specifically on coffee in humans, available in the literature as clinical studies and randomized clinical trials, systematic reviews and meta-analyses, case reports, case series, and editorials. The search excluded articles not available in English or articles published more than 10 years ago. Also excluded were articles that were not peer-reviewed and published in medical journals.

The Cochrane database was searched using the same keywords, yielding 0 results for "coffee neuroplasticity" and "neuroplastic effects + coffee" and two results for "caffeine neuroplasticity," which were not relevant to our research subject. The net result from the Cochrane database search was 0.

A Google Scholar search was conducted for the same keywords, and the first 20 results were considered. The Google Scholar search casts a broad net, but results are page-ranked so that the best matches appear at the top of the results. Google Scholar searches can result in thousands of retrieved items, including patents, book chapters, articles, gray literature, consumer articles, and so on. For that reason, we followed a strategy used by others to take only the first 20 results (two pages). After removing non-peer-reviewed articles, duplicates from other searches, and articles not explicitly related to coffee and neuroplasticity, Google Search yielded a total of 22 results.

This resulted in a total of 109 peer-reviewed articles considered for synthesis in this publication. In addition, we reviewed the bibliographies of relevant articles. All searches were conducted in March of 2025. Because this is a narrative and not a systematic review, evidence was considered from various types of articles (reviews, editorials, and clinical studies), but not all articles retrieved related to one specific research question, which is the case in systematic reviews. As such, it was not possible to grade evidence, since many articles retrieved were not clinical trials or randomized clinical trials.

## Review

Current academic interest in neuroplasticity, the brain’s ability to alter its neural connections in response to environmental stimuli, experiences, learning, injury, and disease processes, has resulted in explorations of such crucial topics as recovery from brain damage, attenuation of neurodegenerative diseases, advances in geriatric medicine, and the physiology of learning and memory [8]. Neuroplasticity is affected by aging. The rapid formation of new neural connections characteristic of childhood becomes more limited, regulated, slower, and situational with age. Adult neurogenesis is not a misnomer; it does occur, but primarily in the subventricular zone of the brain and in the dentate gyrus of the hippocampus, which are brain regions associated with learning and memory [9-11]. Adult neurogenesis may be facilitated by exercise and exposure to enriched environmental stimulation [9,12,13]. The putative role of food for neurologic support has found widespread popular and growing academic interest.

Synaptic plasticity is defined as the activity-dependent modification of the strength and/or efficacy of synaptic signaling from pre-existing synapses. It must be considered within the context of neuroplasticity. Synaptic plasticity has been studied in the context of neural circuitry, memory, learning, neuropsychiatric disorders, and degenerative diseases. It is known that neural networks in mammals can be altered not just by neuroplasticity but also by both adaptive and maladaptive changes in synaptic plasticity [14]. Repeated stimulation of synapses results in long-term potentiation (LTP), a type of functional neuroplasticity [8]. LTP is a crucial component in learning and memory, while its antithesis, long-term depression (LTD), defines a progressive weakening of synapses, linked to deteriorating memory and learning limitations [15].

Human studies of coffee and brain activity

While most studies exploring the effect of coffee on the brain, neurogenesis, neuroplasticity, and synaptic plasticity have been conducted in animal models, several important clinical trials in humans suggest the role of coffee in human brain activity, as summarized in Table 1.

**Table 1:** Studies on the effects of coffee on human brain activity These studies are presented in alphabetical order by surname of the first author [16-30]. Below are abbreviations used in the table. ACTH: adrenocorticotropic hormone, BP: blood pressure, d: day(s), db: double blind, h: hour(s), DHEAS: dehydroepiandrosterone sulfate, EEG: electroencephalography, HPA: hypothalamic pituitary adrenal, iTBS: intermittent theta-burst stimulation, mg: milligram(s), min: minute(s), pt: patient(s), RCT: randomized clinical trial, sAA: salivary a-amylase, vs.: versus, wk: week(s)

Study	Population	Objective(s)	Outcomes	Comments
Alharbi et al., 2018 [16]	300 healthy young women in a db RCT	Comparison of coffee robusta to coffee arabica in terms of results in attention and memory tasks	No significant differences between groups. Both types of coffee conferred benefits on attention, cognitive abilities, and memory. Coffee arabica was significantly superior for addressing sleepiness and improving response times	Arabica and robusta are the two main types of coffee beans sold around the world
Best et al., 2021 [17]	40 healthy adults in a db RCT	Evaluation of a whole coffee fruit extract plus herbal supplements on performance in cognition, memory, and attention	The coffee group performed significantly better in accuracy, response time, neural efficiency, and cognitive performance	Synergistic effects of constituents suggested
Best et al., 2024 [18]	52 healthy adults in a db RCT	Cognition and brain activity at baseline, 28 d postintervention, and 14 d post-washout. Participants took a daily extract of ginseng, bacopa monnieri, and 100 mg of whole coffee fruit extract	Coffee beverage improved the positive effect, increased the speed of delayed recall at 28 d vs. placebo	Synergistic effects of constituents suggested
Boere et al., 2016 [19]	103 university students, RCT	Do stress and caffeine affect cognition through the same pathway, as tested using manipulation vs. an arithmetic task?	Neither caffeine nor stress conferred a benefit to task completion, and there was no indication of interaction between caffeine and stress	Stress and caffeine seem to affect performance, albeit through separate mechanisms
Frick et al., 2021 [20]	40 depressive adults receiving iTBS or sham twice daily for 10-15 weekdays to manage depression	Participants self-reported coffee and energy drink consumption starting 2 d before treatment to identify a possible association with depressive symptoms	Habitual coffee consumption improved symptoms in the iTBS group, but not in the sham groups	Coffee or energy drink consumption appears to enhance iTBS benefits in reducing depression
Haskell-Ramsay et al., 2018 [21]	72 adults who regularly consumed 2 cups of coffee or 3 cups of tea daily in a db RCT with a counterbalanced crossover design	Comparison of regular brewed coffee, decaffeinated coffee, and placebo on cognition and mood, measured by episodic memory, working memory, attention, and subjective state	The regular coffee consumption group showed increased digit vigilance, decreased fatigue, and fewer headaches compared to the decaffeinated coffee group. Compared to placebo but not regular coffee, decaffeinated coffee improved alertness	Suggests that the cognitive, memory, and subjective effects of coffee are not entirely due to caffeine content
Jackson et al., 2020 [22]	32 adults in a db RCT comparing apple, blueberry, or coffee berry extract infused in a beverage, which in all groups contained beets, ginseng, and sage	Comparison of mood, cognition, and cerebral blood flow at baseline, then again at 60, 180, and 360 min after ingestion	The most pronounced effect on mood and cerebral blood flow occurred in those who took the apple or coffee berry drinks; blueberry extract has a less pronounced effect	Either the phenolic extracts (apple and coffee berry) synergistically enhanced the base drink of beet, ginseng, and sage, or the phenolic extracts (apple and coffee berry) were solely responsible for the effects on mood and cerebral blood flow
Jackson et al., 2022 [23]	46 adults in db crossover RCT of individuals consuming 1100 mg coffee berry extract, 1100 mg coffee berry extract plus 275 mg apple extract, 100 mg coffee berry extract plus 275 mg apple extract, or placebo on 4 occasions	Completion of cognitive tests and mood assessments before dose, then again at 1, 3, and 6 h after dose	1100 mg of coffee berry extract had more effects on alertness than on cognition	The addition of apple extract did not appear to work synergistically with the coffee berry extract, as it did not enhance mood
Lin et al., 2024 [24]	36 adults who habitually took <450 mg/d caffeine in 9-lab-day study with restricted sleep and coffee schedule	Imaging study to determine if acute sleep deprivation plus coffee consumption reduces grey matter in human brains	Cortical plasticity occurred independently of caffeine intake. Grey matter plasticity showed regional changes following sleep deprivation, with a paradoxical effect of caffeine. Caffeine decreased prefrontal and thalamic cortices after sleep deprivation, while abstinence from caffeine for 5 d increased grey matter in the temporal-occipital and thalamic cortices	Changes in grey matter volume reduction caused by sleep deficits may be reversed by caffeine
Masdrakis et al., 2015 [25]	19 pt with panic disorder with or without agoraphobia in a db crossover RCT	Pt were challenged with 400 mg caffeine (instant coffee) or a placebo and evaluated for ACTH, cortisol, and DHEAS at baseline and after	47% of pt (n=9) panicked after caffeine challenge, 0% panicked after placebo. ACTH, cortisol, and DHEAS increased significantly in the coffee but not the placebo groups	Caffeine-induced panic attacks are not associated with HPA-axis activation
Pachimsawat et al., 2021 [26]	71 adult pt without dental anxiety at the dentist’s office. About half of the pt were regular coffee drinkers, and 88% liked the smell of coffee	Pt were subjected to coffee or placebo aroma as they underwent dental probing and scaling. Cortisol and sAA levels were measured, and BP and pulse rate were monitored	Coffee aroma pt had significantly lower sAA and cortisol levels, plus lower pulse rates than those who were exposed to the placebo aroma	Frequency of coffee drinking was not associated with sAA or cortisol levels. This is the first study of coffee as aromatherapy. It is not clear what volatile compounds were inhaled from the coffee aroma
Papakonstantinou et al., 2015 [27]	40 adults in a crossover RCT for assessment of BP, sAA, and anxiety	Groups consumed 160 mg caffeine in hot coffee, cold coffee, cold espresso, and hot filtered coffee, 1 wk apart, with salivary samples and psychometrics assessed at baseline and then at various time points up to 180 min post-consumption. BP was measured at the start and conclusion of each intervention	Coffee significantly elevated sAA over baseline but did not affect salivary cortisol or self-reports of anxiety. Coffee increased BP significantly, but within healthy levels. No differences observed among types of coffee drinks	No significant gender effects observed
Robinson et al., 2020 [28]	71 adults with mild cognitive decline in a 4-arm db RCT	Participants took a coffee cherry extract capsule or placebo twice a day and were assessed for reaction time and accuracy doing cognitive challenges; groups were assessed over 1 wk for a total of 28 d	Coffee cherry extract reduced reaction time and trended toward better accuracy vs. placebo	Coffee cherry extract is a proprietary powdered blend of whole coffee cherries from coffee arabica with <1% caffeine
Ullrich et al., 2015 [29]	17 young adults (19-40 years) in a db RCT of cognitive skills	Caffeine and glucose were administered, and subjects were tested for processing speed, numeric and verbal memory, concentration, and mood over a 2-h period. Subjects were given coffee, sugar, water, placebo; the control group had no treatment at all	Neither coffee nor sugar improved cognition vs. placebo, water, or no treatment, but coffee and placebo improved mood and boosted the subject’s self-assessment of performance	The subjective benefits of coffee or placebo on cognition were enhanced if the subject had been abstinent from caffeine for 24 h prior to the test
Weibel et al., 2021 [30]	20 young males who habitually consumed coffee throughout the day assessed for sleep architecture in a db RCT	Subjects took 150 mg caffeine 3 time/d in coffee, or took a placebo, or entered withdrawal. At 9 d, sleep architecture was measured by EEG starting 8 or 15 h after the last intake of caffeine	There were no EEG-observed differences in sleep time, latency, architecture, or subjective assessment of sleep quality among groups	Healthy sleepers who consume caffeine do not suffer adverse impacts on their sleep

Caffeine in coffee

The most studied constituent in coffee is caffeine (1,3,7-trimethylxanthine), which can interact with synaptic surface receptors and chemical modulators. Caffeine activates intracellular calcium while inhibiting adenosine and GABA receptors [31]. Caffeine exerts an ergogenic effect, improving physical endurance and performance. For that reason, caffeine is widely used by athletes to enhance performance, but it is only effective within a narrow window of roughly 3 to 6 mg/kg. Doses that exceed 9 mg/kg are not only unsafe, but they also do not confer any beneficial ergogenic effects [32]. A murine study provides evidence that caffeine’s beneficial ergogenic effects occur because of neuroplastic changes, namely a shift from LTD to LTP in the striatum [32]. LTP strengthens synapses, while LTD weakens them; both play crucial roles in learning, memory, and cognition [33].

Caffeine is one of several purinergic constituents in coffee. Other purine metabolites are theobromine, theophylline, and paraxanthine [4]. By stimulating neural oscillators in the cortex, caffeine may create pathways that facilitate neuroplastic changes and enhance communication between cortical regions, which depend on N-methyl-D-aspartate receptors. In other words, caffeine appears to have the ability to reorganize the cortical networks [31]. Caffeine pathways within the brain may enable neuroplastic modifications to become more efficient. Most of the limited work on coffee and neuroplasticity focuses on caffeine at doses higher than those consumed by most ordinary coffee drinkers, whose daily caffeine intake falls in the low micromolar range [5].

Caffeine and adenosine

Caffeine has a complex relationship with adenosine, the four adenosine receptors (A1, A2A, A2B, and A3), and the related molecule, adenosine triphosphate (ATP). Caffeine and adenosine share a chemical structure so similar that both substances bind to adenosine receptors [34]. Caffeine may be considered a competitive antagonist of adenosine. Although caffeine is not a selective inhibitor of adenosine, it attenuates frequency-induced LTP in the hippocampus in a way that is more congruent with A2A blockade [5].

Adenosine is closely related to ATP. While coffee acts as an adenosine receptor blocker, it does not inhibit ATP receptors [31]. When ATP is hydrolyzed, energy is released. Adenosine is a component of RNA and acts as a signaling molecule. Adenosine has been described as a neuromodulator that acts as a homeostatic transcellular messenger [35]. However, adenosine cannot be considered a typical neurotransmitter [36]. Presynaptic neurons store ATP in the vesicle, but during the action potential, the vesicle fuses with the presynaptic membrane of the presynaptic neuron. In other words, ATP does not convey information unidirectionally from the presynaptic to the postsynaptic components. The result is that both glutamate and ATP are released, with ATP converting to adenosine by the enzymatic action of ectonucleotidase. This newly released adenosine can bind to presynaptic A1 and A2A receptors, resulting in an inhibitory effect. However, it may also bind to the A2A postsynaptic receptor, which paradoxically has an excitatory effect [36].

Adenosine is an endogenous modulator of the central nervous system, and high levels of adenosine can serve as a biomarker for high rates of neuronal activity, such as might occur during ischemia. Adenosine can help restore the balance between energy consumption and energy availability, leading to its description as a “retaliatory metabolite” [37]. Adenosine has also been studied as an anti-inflammatory agent, an anticancer agent, and a factor in bone remodeling and sleep architecture. It may have analgesic properties along with cardiovascular benefits and neuroprotective effects [36].

Extracellular ATP in striatal brain circuits is an important but unexplained phenomenon associated with Parkinson’s disease. ATP is a prominent extracellular signaling molecule and can engage the ligand-gated ion channels known as P2 receptors [35]. These P2 receptors are the P2XR (ionotropic receptors) and the P2YR (metabotropic receptors). Metabotropic receptors, also known as G-protein-coupled receptors, trigger intracellular events via signaling pathways rather than opening ion channels; this capability can launch a cascade of intracellular activity [38].

Adenosine itself is a neuromodulator that acts on the brain by inhibiting the A1 receptors but facilitating the A2 receptors [39]. A1 receptors are involved in basal synaptic transmission, but ATP released from the synapses may be associated with synaptic plasticity and learning. A2 receptor blockers can reduce behavioral and neurochemical aspects of Parkinson’s disease in a rat model [40]. It remains unclear whether the over-functioning of A2 receptors contributes to the motor dysfunction of Parkinson’s disease or if it is a maladaptive response to motor dysfunction [35]. Likewise, ATP production plays a role in the pathology of Alzheimer’s disease [41]. Its recently elucidated ability to inhibit protein aggregation in the brain has made ATP a potential target in drug development for many types of neurodegenerative disorders [42].

Caffeine and cognition

Nearly all of the caffeine in coffee (99%) is absorbed in the stomach and small intestine within 45 minutes after ingestion [43]; it is metabolized via the cytochrome (CYP) 1A2 enzyme to mono-methylxanthine, dimethylxanthine, and methylated uracil derivatives [44]. Caffeine inhibits lipid peroxidation and can reduce the production of reactive oxygen species (ROS), thereby potentially reducing oxidative stress [45]. Caffeine enhances the activity of glutathione S-transferase and can prevent derangement and apoptosis of red blood cells [46]. Caffeine scavenges hydroxyl radicals, which support its neuroprotective effects [1].

The cognitive benefits of coffee consumption were reported in the Three City Study, a four-year population-based study from France of 4,197 women and 2,820 men [47]. This is one of the few studies that found sex-based differences for coffee drinkers, notably that coffee conferred more pronounced benefits to women. Women who consumed more than three cups of coffee per day showed significantly less decrease in verbal retrieval and visuo-spatial memory compared to women who had one cup or less of coffee per day. However, a similar effect did not occur in men. Moreover, the protective benefits of caffeine increased with age in women, but not in men [47]. Similar findings were reported from a study in Portugal of 648 participants ≥65 years, which reported that caffeine intake of >62 mg/day was associated with significantly less cognitive decline than <22 mg/day, but only in women; there was a numeric but not statistically significant lowered risk for men [48]. Although not well studied, it has been suggested that endogenous hormones may play a role in caffeine’s effects on women [1].

In rodents, administering caffeine in drinking water at concentrations of 1 g/L has been effective in providing neuroprotection by shielding the animals from noxious brain injuries that serve as preclinical models for Alzheimer’s disease, diabetes, stress, and juvenile convulsions [5]. In a study of 50 µm hippocampal slices obtained from mice, caffeine facilitated synaptic transmission by 40% while, at the same time, decreasing paired-pulse facilitation and LTP amplitudes (a metric of synaptic plasticity) [49]. In this study of rats administered 1 g/L of caffeine daily for three weeks, the hippocampus regions of their brains exhibited a concentration of 22 µm of caffeine, which attenuates LTP [49]. While caffeine is known as a nonselective adenosine receptor antagonist, its effects on hippocampal slices appear similar to those produced by selective adenosine receptor antagonists. Furthermore, animal studies have shown that these benefits do not diminish with age [5]. In humans, caffeine ingested in foods and beverages can block both central and peripheral adenosine receptors [50].

Caffeine improves cognitive performance for simple and complex tasks, but there is only equivocal evidence for a dose-dependent relationship between caffeine and improved cognitive performance. It is also not clear if regular caffeine consumption enhances cognition more than occasional caffeine use [51].

Caffeine may alter brain morphology. In a study of 45 healthy young women aged 19 to 30, both high-level and low-level caffeine intake from coffee consumption were associated with a larger hippocampus brain area compared to moderate-level caffeine intake from coffee. No other areas of the brain were similarly affected [52]. While it has been suggested that coffee-induced changes in the brain were temporary and can be reversed over time if the person no longer consumes caffeine, habitual use of coffee has been implicated in persistent changes in neural networks [53].

Other coffee constituents

Polyphenols

Much of the research involving coffee focuses on caffeine. Yet, coffee is rich in a variety of constituents that likely play a role in its effects, whether independently or by additive or synergistic effects. These include polyphenols, a large category of phytochemicals. Diets rich in polyphenols are believed to improve neuroplasticity and help protect against neurodegeneration [54]. Polyphenols can affect biochemical reactions in the body and are one of the most abundant sources of antioxidants in the food supply. They can be roughly categorized as flavonoids (isoflavones, quercetins, cyanidins, and catechins) and phenolic acids (caffeic and ferulic acids), and their neuroprotective and anticancer benefits are the subject of considerable study [55]. Note that caffeic acid is not related to caffeine.

Cholinergic Acid

The cholinergic system forms a communication link between the central and peripheral nervous systems. It works with acetylcholine (Ach), its receptors (AchRs), and the enzymes choline acetyltransferase (ChAT) and acetylcholinesterase (AChE). Dysregulation of this system can be the prelude to maladaptive inflammatory processes and autoimmune disorders [56].

Cholinergic acids are esters formed between trans-cinnamic acids and quinic acid. Cholinergic acids possess antioxidant properties that appear to reduce oxidative stress and, in turn, decrease neuroinflammation [7, 57]. Cholinergic acids are present in coffee, although research into these specific coffee constituents as individual substances has been limited [58]. While an animal study found no evidence that cholinergic acids had any direct effect on synaptic activity in the brain, an in vitro model of ischemia showed that chlorogenic acids could aid in the recovery from deteriorating synapses [58].

Chlorogenic Acid

Chlorogenic acid is a non-flavonoid polyphenol found in coffee and numerous other fruits, vegetables, wine, tea, and olive oil. Both caffeinated and decaffeinated coffee are rich in chlorogenic acids (70 to 350 mg per cup) [7, 59]. Chlorogenic acid has anti-inflammatory, cardioprotective, chemopreventive, anti-diabetic, and anti-obesity effects and also protects the liver [7]. Despite preclinical evidence, its role in human neuroprotection and cognitive aid is less well documented [7]. A review of the biologic properties of chlorogenic acid in coffee speculated that it could play crucial roles in lipid and glucose metabolism and offer health benefits in numerous other conditions, including cancer, heart disease, and diabetes [60].

Caffeic Acid 

Despite its name, caffeic acid is not related to caffeine; it is a specific type of polyphenol. Caffeic acid is a hydroxycinnamic acid with powerful antioxidant effects, surpassing those of other constituents in coffee [61]. Caffeic acid is present in various foods and is a well-known constituent of red wine and tea. Preclinical studies suggest that caffeic acid may benefit cognition, reduce Aϐ plaques, and improve neurogenesis within the hippocampus [62]. In a rat model of Alzheimer’s disease, caffeic acid significantly improved learning deficits and boosted cognitive function compared to a placebo, while simultaneously reducing oxidative stress and inflammation [63].

Trigonelline

Trigonelline is an abundant alkaloid found in coffee beans. Despite its well-documented antiviral, antibacterial, anti-tumor, and anti-hyperlipidemic activity, it has not been as thoroughly investigated as many of the other constituents in coffee [64, 65]. Related to niacin, trigonelline may account for up to 1% of the dry matter in roasted coffee beans [66]. In preclinical studies, trigonelline has shown neuroprotective effects, likely resulting from reduced oxidative stress, inhibition of pro-inflammatory cytokines, and its ability to regulate and restore brain-derived neurotrophic factor [67].

Pretreatment with trigonelline in an in vitro study was able to reduce oxygen-glucose deprivation/reperfusion-induced injury to the neurons of the hippocampus, reducing ROS and decreasing the concentration of superoxide dismutase and glutathione peroxidase [64]. Further in vitro studies show that trigonelline has antiglycation properties, possibly associated with neuroprotection. This is of particular clinical relevance, since advanced glycation end products may contribute to the amyloidosis characteristic of Alzheimer’s disease. Murine studies suggest that glycoxidation plays a role in the pathogenesis of Alzheimer’s disease [7].

Diterpenes

Cafestol is a diterpene most plentiful in coffee arabica and, to a lesser extent, in other coffees. Cafestol is extracted during the brewing process and is more abundant in unfiltered coffee drinks, such as espresso and French-press coffee. Kahweol is a diterpene derived from cafestol [68] and promotes mitochondrial protection [69].

Other Substances

Phenylindanes are formed during the roasting of coffee when high temperatures break down chlorogenic acid lactones. They are believed to inhibit the aggregation of Aϐ and Γ proteins in the brain associated with Alzheimer’s disease [70]. An early study found oral phenylindanes had good bioavailability and could readily cross the blood-brain barrier [71].

Eicosanoyl-5-hydroxytryptamide (EHT) is a natural fatty acid derivative of serotonin found in coffee beans, which modulates protein phosphatase (PP2A) methylation in murine models of Alzheimer’s disease and Parkinson’s disease [72, 73]. EHT is not related to caffeine. In a rodent study, animals were exposed to elevated levels of soluble oligomeric Aϐ, associated with the development of cognitive impairment in Alzheimer’s and Parkinson’s disease. EHT protected the animals from cognitive impairment as evidenced in a radial-arm water maze test and a task based on the development of a contextualized fear. Further, acute and chronic administration of EHT prevented Aϐ-associated impairments for an extended period [73].

EHT can interact with PP2A, a heterotrimeric protein made up of a structural subunit (A), a regulatory subunit (B), and a catalytic subunit (C) [74]. It belongs to the family of Ser/Thr phosphatases and is involved in cellular signaling and other physiologic processes; malfunction of these proteins has been linked to pathological processes. The phosphorylation of regulatory proteins in normal cells is primarily coordinated by the actions of Ser/Thr protein kinases and Ser/Thr protein phosphatases [74]. In humans, there are over 95 complete, functional, and catalytically active enzymes (holoenzymes or conjugate enzymes) that are built from just two catalytic subunits [75]. Impaired or dysregulated PP2A methylation is believed to increase the risk of Alzheimer’s disease via hyperhomocysteinemia, an abnormally high level of serum homocysteine [76].

Coffee in specific neurodegenerative conditions

Coffee, in particular caffeine, has demonstrated highly beneficial effects on neurocognitive performance and seems to reduce the risk of neurodegenerative diseases. In this context, we must consider that numerous factors can modulate individual outcomes, including age, sex, genetics, and comorbidities, necessitating personalized guidance. Much of the evidence favoring coffee in specific health conditions comes from small, observational studies rather than rigorous randomized controlled trials [77]. Nevertheless, certain specific conditions remain of interest in this context.

Note that in all of these studies, the relationship between coffee and protection against specific conditions is, at most, correlational and not causal. Further, in some cases, the neuroprotective benefit may be a delay in the onset of symptoms rather than prevention.

Alzheimer’s Disease

The most common neurodegenerative disorder is Alzheimer’s disease, which is likely to increase in prevalence as global populations age [78]. The global prognosis for Alzheimer’s prevalence is grim. Animal studies suggest that Alzheimer’s disease may be delayed with caffeine or coffee, but evidence from murine studies cannot be readily generalized to human subjects. Alzheimer’s disease is multifactorial, and genetics and environmental influences also play a role in pathogenesis. While genetics plays a role, the familial form of the disease accounts for <1% of all cases, and the sporadic form accounts for the rest. However, from a histopathological perspective, these two forms are identical [72].

Like Parkinson’s disease, Alzheimer’s disease is a tauopathy, a type of neurodegenerative disorder associated with dementia [79]. Alzheimer’s disease is by far the most prevalent of such disorders and the leading cause of dementia, representing a global public health crisis [80]. Tauopathies involve the aggregation and spreading of tau proteins.

Alzheimer’s disease occurs with the accumulation of misfolded αϐ and tau proteins in amyloid plaques and neuronal tangles within the brain [81]. Coffee consumption was associated in a nonlinear relationship with reduced risk of dementia [82], but studies of the beneficial effects of coffee on Alzheimer’s disease have produced mixed and contradictory results. Animal studies have been more promising, but it is not clear if these results can be generalized to humans [83]. Caffeine in coffee may be more accurately termed a “cognitive normalizer” instead of a “cognitive enhancer” [80].

A pooled analysis of four observational studies found that coffee consumption is inversely associated with the risk for developing Alzheimer’s disease [84]. Despite positive results from preclinical studies, human studies are obviously still needed. Although caffeine is metabolized along similar pathways in rodents and humans, caffeine metabolism in humans takes place mainly by way of N-3 demethylation, producing paraxanthine (1,7-dimethylxanthine), theobromine (3,7-dimethylxanthine), and theophylline (1,3-dimethylxanthine), of which a small amount is converted to 1,3,7-trimethyluric acid by way of C-8 hydroxylation [85]. Rodents metabolize caffeine mainly by C-8 hydroxylation to produce several trimethyl derivatives, of which N-1 theobromine and N-7 theophylline are the most notable [86]. Extensive research on caffeine has relied on the rodent model, which is a limitation [4].

A systematic review and meta-analysis found that people who drank one or two cups of coffee per day, as well as people who drank two to four cups of coffee per day, had a statistically significantly lower risk of developing Alzheimer’s disease than those who did not (risk ratio 0.68, p=0.0). However, people who drank more than four cups of coffee a day had a slightly increased risk of developing Alzheimer’s disease (risk ratio 1.04, p=0.0) [87]. In fact, moderate coffee intake, defined as two to four cups per day, compared to no coffee consumption, has additional benefits, including a reduced risk of all-cause mortality [88].

Parkinson’s Disease

Parkinson’s disease is characterized by the degeneration of dopaminergic neurons in the substantia nigra of the basal ganglia, leading to motor symptoms. Potential pathways may include any or a combination of the following: oxidative stress, mitochondrial disruption, and neuroinflammation. While coffee has emerged as a potential protective factor for Parkinson’s disease, the role of diet in general remains controversial. This is because there are likely genetic, microbiota, and environmental factors that come into play [89], the influence of which has not yet been reliably quantified. A variety of potentially protective factors for Parkinson’s disease have been named, including tobacco use, physical activity, nonsteroidal anti-inflammatory drugs (NSAIDs), calcium channel blockers, and statins. At the same time, pesticide exposure, traumatic brain injury, lead exposure, and air pollution are considered risk factors [90]. The potential benefits from coffee are likely imparted by caffeine and would thus also occur with the use of tea [90].

An umbrella review of 59 unique outcomes of observational studies and randomized controlled trials reported a “probable decrease” in Parkinson’s disease associated with both coffee as a beverage and caffeine in general [91]. Caffeine’s ability to inhibit the A2A adenosine receptor may play a role in its neuroprotective benefits with respect to Parkinson’s disease; the A2A receptors are densely expressed in dopamine-rich portions of the brain [92]. The circumscribed distribution of A2A receptors has increased interest in A2A blockers as potential drug targets, which have shown promise in animal studies [93]. Adenosine can decrease the neurotransmission of dopamine, so A2A blockade would therefore promote dopaminergic transmission [93]. The loss of dopamine in Parkinson's patients can cause GABA-stimulated inhibition of excitatory glutamate signaling, which can lead to bradykinesia and akinesia. Adenosine can stimulate GABA signaling along the A1 and A2A receptor pathways, while caffeine can inhibit GABA signaling [94].

A case-control study of 1,208 participants did not find any associations between coffee consumption and susceptibility to Parkinson’s disease [95]. On the other hand, a recent meta-analysis reported that coffee consumption, specifically caffeine consumption, could reduce the risk of development of Parkinson’s disease and retard its progression in those already affected [96]. A study of 304,980 participants conducted by the National Institutes of Health reported that coffee had a mild but demonstrable neuroprotective effect against Parkinson’s disease [97]. Coffee consumption, as well as the intake of other caffeine-containing products, has been credibly but not consistently linked to reduced risk of Parkinson’s disease [1,7].

Coffee appears to confer greater benefits on men than women in terms of protection against Parkinson’s disease, and women taking hormone replacement therapy derive no discernible neuroprotective benefit from coffee [90]. There may be a dose-response effect in this context, with maximum protection thought to be optimally achieved with approximately three cups of coffee per day [98].

Stroke

Stroke and post-stroke dementia are increasing in prevalence and represent a substantial healthcare burden. In a prospective cohort study of 365,682 participants between the ages of 50 and 74 years, drinking coffee and/or tea was associated with a reduced risk of stroke and dementia, and coffee intake (whether coffee only or coffee plus tea) could be linked to a lower risk of post-stroke dementia [99]. Drinking two to three cups of coffee per day was associated with the lowest hazard ratio for incident stroke and dementia [99]. However, the case-controlled INTERSTROKE study (n=13,462 cases and 13,488 controls) found that drinking five or more cups of coffee per day was associated with a higher risk for ischemic stroke and all strokes. In contrast, lower intake (four or fewer cups) had no association with stroke. On the other hand, tea consumption was also studied in INTERSTROKE and found to be associated with reduced risk of stroke, even at high consumption rates [100].

Analgesic benefits of coffee

Caffeine is a well-established adjuvant analgesic contained in many over-the-counter pain relievers. Caffeine enhances the bioavailability of analgesics, such as acetaminophen or ibuprofen, which allows for a more rapid onset of analgesic action. The role of caffeine on the central nervous system is more complex due to its action on adenosine receptors, which can block nociceptive signaling. Caffeine acts as a vasoconstrictor and can reduce inflammatory pain [101].

Pain can impair cognition, depress mood, hamper memory, and compromise executive function. Caffeine may promote better cognition in pain patients [102]. The effects of chronic pain on cognition occur through multiple distinct pathways. They are associated with structural and functional changes in the brain, particularly in the dorsolateral prefrontal cortex, the medial prefrontal cortex, and the default mode network [102]. Since the relationship between cognitive deterioration and chronic pain is bidirectional, it is speculated that age-related cognitive decline may contribute to chronic pain or vice versa [102]. The role of caffeine in terms of memory enhancement is equivocal [50], but its cognitive benefits are well established [51]. In other words, caffeine may help mitigate impaired mental states induced by chronic pain.

Discussion

Despite animal studies, epidemiologic analyses, and observational clinical studies supporting the cognitive benefits of coffee consumption, the neuroprotective benefits of coffee in humans have yet to be fully elucidated [103]. Although a 30-year study of 137 men found an inverse association between Parkinson’s disease and coffee consumption, the exact constituents of coffee associated with neuroprotection and their mechanisms remain unclear [104]. Other studies have produced mixed results.

Coffee is a complex natural substance, and it is possible, even probable, that there is an additive or synergistic interplay among the many substances in coffee that contribute to these observed neuroprotective effects [68]. Coffee may modify brain volume, change neuronal connections, and alter synapses by building new pathways or rerouting old ones. Its potential neuroprotective benefits and anti-neuroinflammatory effects make it an important area of study, especially given the increase in neurodegenerative diseases among aging populations. Furthermore, coffee may help reverse or buffer cognitive deficits resulting from chronic pain and is widely used as an analgesic adjuvant [101]. Genetic effects and sex-specific responses to coffee further complicate the study of coffee.

It may be argued that coffee, or any food or beverage, is best considered within the context of a broader Mediterranean diet, which emphasizes fruits, vegetables, whole grains, fish, and olive oil, while limiting meat and avoiding processed foods. Coffee as part of such a diet may provide benefits, but it would be complicated, if not impossible, to sort out which benefits could be traced to which specific foods. Overall, the Mediterranean diet is associated with demonstrable and sustainable health benefits, and coffee may be incorporated into this eating plan [105]. It is not known if coffee, or indeed any food product taken individually, confers the same health benefits as the overall Mediterranean diet or other recognized healthful diets.

The dose relationship of coffee’s benefits is still being studied. In the 22-year HUNT clinical study (n=7,381), coffee drinking was not associated with dementia risk in any dimension except at high consumption rates; women who drank eight or more cups of boiled coffee daily had an increased dementia risk, but men who drank four or five cups of coffee a day had a decreased risk of dementia [106]. Such studies are confounded by many factors: what constitutes “a cup of coffee,” and do the type of coffee bean, roasting process, or coffee preparation affect the benefits?

In contrast to numerous studies reporting potential neuroprotective benefits from coffee, a meta-analysis found that coffee consumption had no association with the risk for developing Alzheimer’s disease in a total of 36,300 patients [107]. These mixed results make it challenging for clinicians to counsel patients with respect to coffee consumption. Beyond Alzheimer’s and Parkinson’s diseases, affective disorders, such as depression, anxiety, or bipolar disorder, have been associated with deficits in neurogenesis and synaptic plasticity and imbalances among neurotransmitters. This suggests that coffee or possibly other caffeinated beverages such as cocoa or tea may attenuate these conditions, but high-quality randomized clinical trials supporting such claims are lacking [108].

Overall, the close connection between diet and health has long been recognized by medicine, although it has not always been thoroughly studied. Hippocrates said that food should be medicine, and coffee is perhaps an example of a beneficial food. The ability to utilize food in this way remains a challenge, since diet is influenced by culture, social context, availability of products, affordability, and seasonality. The ability of the body to utilize beneficial foods depends not only on the food itself but also on the cooking method, added ingredients, freshness, and individual genetics and microbiome. The integration of coffee into a healthful diet, such as the Mediterranean diet, is not contraindicated by our limited and equivocal knowledge; coffee does not appear to harm.

This study has significant limitations. First, it is a narrative review, not a systematic review, offering a broad overview of various topics related to neuroplasticity, neurocognitive function, and coffee, rather than an in-depth analysis of any specific aspect. We acknowledge that the literature on the topic is both heterogeneous and equivocal, and studies were not ranked based on the quality of evidence. Most of the available data come from observational studies rather than extensive and rigorous randomized clinical trials. There are numerous confounding factors in any study of coffee, such as the role the specific coffee constituents may play. Different types of coffee beans, roasting processes, and preparation methods could influence results.

Additionally, there are multiple important constituents in coffee that quite likely play a role in its putative properties. We have considerable data from animal studies, but rodent studies, particularly those addressing caffeine metabolism, are not necessarily generalizable to humans. Finally, caffeine metabolism can be affected in humans by genetic polymorphisms, such as those along the CYP-1A2 substrate. Most coffee studies, including the ones evaluated in this paper, do not control for this. While a systematic review might seem helpful, the body of current literature on coffee studies is so vast and heterogeneous that such an approach would be impractical, if not impossible.

## Conclusions

Coffee is a natural, widely consumed, and complex natural product whose regular use has been linked to protection against neurodegenerative disorders, in particular Alzheimer’s and Parkinson’s diseases. It is not clear if the benefits of coffee are derived from caffeine or any of the other components of coffee, such as its numerous phytochemicals, and it is not unreasonable to think coffee's benefits may involve an additive or synergistic interplay of multiple substances. The evidence suggests that coffee may offer neuroprotective benefits, improve cognition and memory, alter brain morphology, and enhance attention. Given that chronic pain is known to impair cognitive function, coffee’s mild analgesic benefits may also contribute indirectly to cognitive preservation. These complementary pathways highlight coffee’s complex, multimechanistic influence on the aging brain. With our graying population, these potential neuroprotective effects of coffee warrant further study.
